# Socio-Demographic and Health Determinants of Overnutrition in Hungarian Women Aged 65 Years and Older

**DOI:** 10.3390/nu17243836

**Published:** 2025-12-08

**Authors:** Salome Zurashvili, Amr Sayed Ghanem, Battamir Ulambayar, Marianna Móré, Attila Nagy

**Affiliations:** 1 Department of Epidemiology, Faculty of Health Sciences, University of Debrecen, 4028 Debrecen, Hungary; salomezurashvili@outlook.com (S.Z.); aghanem@etk.unideb.hu (A.S.G.); ulambayar.battamir@etk.unideb.hu (B.U.); 2 Department of Integrative Health Sciences, Institute of Health Sciences, Faculty of Health Sciences, University of Debrecen, 4028 Debrecen, Hungary; more.mariann@etk.unideb.hu

**Keywords:** overnutrition, older women, lifestyle factors, European Health Interview Survey, Hungary

## Abstract

**Background/Objectives:** Overnutrition, defined as the excessive intake of energy and nutrients leading to increased body mass, is a major contributor to morbidity and mortality worldwide. In Hungary, dietary and lifestyle habits, combined with rapid population aging, make older adults particularly vulnerable to excess weight. This study aimed to assess the prevalence and determinants of overnutrition among Hungarian women aged 65 years and older, focusing on socio-demographic, lifestyle, and health-related factors. **Methods**: A repeated cross-sectional analysis was conducted using pooled data from the 2009, 2014, and 2019 Hungarian waves of the European Health Interview Survey (2385 women aged ≥65 years). Overnutrition was defined as Body Mass Index (BMI) ≥25 kg/m^2^. Associations with explanatory variables were assessed using chi-square tests and multivariable logistic regression, with adjusted odds ratios (ORs) and 95% confidence intervals (CIs). **Results**: The prevalence of overnutrition was highest in 2014 (35.7%) and lowest in 2019 (30.9%). Significant predictors included marital status (OR = 0.77, 95% CI: 0.63–0.94), tertiary education/primary (OR = 1.62, 95% CI: 1.18–2.22), and income level (2nd /1st quintile: OR = 0.67, 95% CI: 0.51–0.88). Smoking, diabetes, arthrosis, and hypercholesterolemia were associated with lower odds. Poor self-perceived health (OR = 1.45, 95% CI: 1.15–1.82) and mental illness (OR = 1.46, 95% CI: 1.05–2.05) were independently associated with increased risk. **Conclusions**: The high prevalence of overnutrition in older Hungarian women highlights the importance of targeted public health strategies addressing social disparities and lifestyle behaviors.

## 1. Introduction

Overnutrition, a form of malnutrition, refers to excessive energy and nutrient intake that increases body fat and is evaluated by Body Mass Index (BMI), with 25.0–29.9 kg/m^2^ classified as overweight and ≥30 kg/m^2^ as obesity [1,2]. In 2022, the World Health Organization reported that about 2.5 billion adults worldwide, 43% of the global population, were overweight, including more than 890 million with obesity [2]. In the European region, nearly 60% of adults fall into these categories [2]. Excess body weight is a major contributor to morbidity and mortality, as it is strongly associated with non-communicable diseases such as type 2 diabetes, cardiovascular disease, hypertension, and several types of cancer [3]. At the individual level, obesity is associated with psychological challenges such as depression and anxiety, while on a larger scale, it creates substantial direct and indirect costs, placing a heavy burden on healthcare systems and social resources [4,5].

The increasing prevalence of overnutrition has been mainly shaped by the “nutrition transition,” characterized by higher consumption of ultra-processed, calorie-rich foods and sugar-sweetened beverages [4,6]. Sedentary lifestyles associated with urbanization, mechanization, and extended screen time further contribute to weight gain [7]. Disparities in socioeconomic status also have a significant impact, as differences in income, education, and access to resources greatly affect food choices, physical activity options, and, in turn, obesity risk [8].

Aging leads to physiological, nutritional, and dietary changes such as decreased muscle mass, slower metabolism, and fat redistribution, raising the risk of excess weight and related comorbidities [9]. Sarcopenic obesity, the combination of muscle loss and fat accumulation, has particularly adverse effects, being linked to frailty, mobility issues, disability, and increased mortality [10,11]. Beyond these biological changes, postmenopausal hormonal decline following menopause, a decrease in lean mass, financial limitations, caregiving responsibilities, and psychosocial stressors all contribute to the increased risk of obesity observed among older women [12]. In Europe, the prevalence of obesity reaches its maximum between the ages of 65 and 74, with females consistently showing higher rates than males [13]. This disparity is especially evident in Central and Eastern Europe, where Hungary is classified among the most affected nations [14].

In Hungary, dietary and lifestyle patterns fall short of European recommendations, as indicated by national surveys reporting high intakes of saturated fats and sugars, frequent consumption of processed foods, and low consumption of fruits and vegetables [6,9]. These patterns reflect broader trends in Central and Eastern Europe associated with the nutrition transition [6]. Meanwhile, Hungary is experiencing rapid demographic aging, with women aged 65 or older among the fastest-growing groups [13]. The coexistence of aging and obesity makes the prevention and management of overnutrition in older women a medical concern and a pressing public health priority [15]. Also, the economic burden is expected to increase as it leads to higher healthcare utilization and costs [16]. Despite this burden, research on obesity among older women in Central and Eastern Europe is still limited.

Addressing this gap requires understanding the multifactorial nature of overnutrition, shaped by personal and social factors beyond a country’s economic status or overall health trends [4]. Lower socioeconomic status, limited income, and lower educational attainment are consistently associated with poorer diet quality and higher obesity rates across Europe [17]. The living environment further influences these disadvantages, as individuals residing in rural areas often have limited access to high-quality food and healthcare services, whereas urban populations are more likely to experience sedentary lifestyles [18]. Relationship status influences weight regulation by shaping eating behaviors and routines, as those living alone are less motivated to prepare healthy meals, resulting in less diverse, lower-quality diets [19].

Smoking and alcohol use are longstanding lifestyle behaviors in Hungary, with high prevalence reported across multiple population groups [20]. National trend data indicate that smoking rates among Hungarian women have increased in recent decades, reflecting broader social and behavioral influences on tobacco use [20,21]. Alcohol consumption is rooted in cultural traditions where regular intake of alcoholic beverages often occurs alongside energy-dense meals, contributing to excess calorie intake and poorer diet quality [9,22].

Socioeconomic, dietary, and cultural factors influence the distribution of chronic conditions [23]. Cardiovascular diseases remain a major public health issue, while diabetes affects 9.1% of adults, with type 2 increasing sharply with age [24,25]. Traditional energy-dense dietary habits have been associated with higher hypercholesterolemia rates, raising cardiometabolic risk and contributing to excess weight [26]. Gastrointestinal conditions like peptic ulcer disease can affect nutritional status through medication use and dietary changes, altering the risk of under- or overnutrition depending on severity and treatment response.

Emotional and psychological factors further elevate the risk of overnutrition. Depression affects about 35.1% of European women aged 65 and older and contributes to emotional eating [27,28]. Additionally, reporting self-perceived health offers vital insight into overall well-being, as lower health status typically indicates a combination of chronic conditions, functional limitations, and psychological distress [29].

Considering the gaps in existing research on older female populations, this study aimed to assess the prevalence and determinants of overnutrition among Hungarian women aged 65 years and older using data from three nationally representative surveys. The analysis investigated how sociodemographic, lifestyle, and health-related factors were associated with overnutrition. By focusing on a highly vulnerable yet underrepresented population, this research aims to generate evidence that can inform targeted prevention and intervention strategies.

## 2. Materials and Methods

### 2.1. Study Design and Data

The study employed a repeated cross-sectional design, using anonymized secondary data from the Hungarian waves of the European Health Interview Survey (EHIS), conducted in 2009, 2014, and 2019. This survey is a standardized initiative coordinated by Eurostat aimed at collecting comparable health data across European Union member states from nationally representative samples of individuals aged 15 and older. The three survey waves align with Hungary’s official EHIS timeline, enabling a reliable trend analysis over ten years. All data collection processes were conducted voluntarily and adhered to both national and European Union ethical standards, with informed consent obtained from all participants. For this analysis, the three datasets were merged to focus on women aged 65 and older, resulting in a final sample of 2385 participants (n = 662 in 2009, n = 757 in 2014, and n = 966 in 2019).

### 2.2. Variables

The primary outcome variable of the study was overnutrition, measured using the body mass index (BMI), calculated as weight in kilograms divided by height in meters squared (kg/m^2^), based on participants’ self-reported values collected through the EHIS questionnaire [30]. According to WHO guidelines, BMI was categorized into normal weight (<25 kg/m^2^) and overnutrition (≥25 kg/m^2^) [2]. Suppose a participant was unable to recall their exact height or weight; an estimated value was recorded. If no reliable estimate was available, the data were marked as missing and excluded from the analysis.

Explanatory variables included socio-demographic, lifestyle, and health-related factors. Marital status was categorized as single (including divorced or widowed) or not single (married or cohabiting), based on participants’ self-reports. Educational attainment was defined at three levels: primary (completion of 8 years of basic education), secondary (completion of high school with a diploma), and tertiary (college or university degree or higher). Household income was measured as equivalized disposable income, calculated using the EU-SILC equivalence scale, and divided into quintiles from the 1st (lowest) to the 5th (highest) [31]. Because income alone may not fully reflect financial strain in older adults, subjective financial status was included as a complementary measure and assessed through self-reported perceptions of their economic situation, which asked participants how easily their household could meet its monthly expenses [30]. Responses were provided on a five-point Likert scale and were categorized into three groups: good (very easily/easily), average (with some difficulty), and poor (with difficulty/great difficulty). The residential area was classified based on the EHIS Degree of Urbanization: urban (cities, towns, and suburbs) or rural (sparsely populated areas) [30]. Lifestyle factors included smoking and alcohol consumption behaviors. Smoking status was assessed with a question about current tobacco use (smoker/non-smoker). Alcohol consumption was determined by asking participants if they had consumed any alcoholic beverages in the past 12 months (drinker/non-drinker).

Mental and self-perceived health were included as psychosocial indicators of emotional well-being and subjective health assessments. The EHIS item evaluated self-perceived health by asking, “How is your health in general?” with five response options, which were recoded into categories of good (very good/good), average (fair), and poor (bad/very bad) [30]. Additional health conditions were recorded as binary variables (yes/no), including diabetes, peptic ulcer, hypercholesterolemia, cardiovascular disease, arthrosis, and mental illness.

### 2.3. Statistical Analysis

Statistical analyses were performed to summarize participant characteristics and identify factors associated with overnutrition. Explanatory variables were selected based on previous research and established literature across four domains. Socioeconomic indicators reflected structural determinants affecting living conditions, resource availability, diet quality, and access to healthcare. Behavioral factors were analyzed due to their effects on metabolic processes, energy balance, and culturally shaped behaviors. Health-related characteristics covered common chronic conditions among adults that may influence body weight through physiological changes or medication use, and psychosocial factors were considered to capture subjective health perceptions and mental health status, as well as their potential effects on eating behavior. The analysis was based on the hypothesis that overnutrition, defined as BMI ≥ 25 kg/m^2^, is associated with sociodemographic, behavioral, health-related, and psychosocial factors among Hungarian women aged 65 years and older.

To examine these associations, descriptive statistics were first used to categorize participants by BMI and calculate frequencies and percentages, with group differences assessed using chi-square tests. Associations between overnutrition and the explanatory variables were explored through multivariable logistic regression. All explanatory variables were entered concurrently into the models to obtain fully adjusted odds ratios (ORs) with 95% confidence intervals (CIs). Separate regression models were estimated for each survey year (2009, 2014, and 2019) and for the combined dataset to evaluate overall trends. Participants with missing BMI or covariate data were excluded. All analyses were performed using Stata IC version 18.0 [32], with statistical significance defined as *p* < 0.05.

## 3. Results

### 3.1. Descriptive Characteristics of the Study Population

Table 1 summarizes the socio-demographic and health-related characteristics of the 2385 Hungarian female participants aged 65 and older, focusing on their BMI status. Overall, 33.1% (n = 783) of the study population were classified as overnourished (BMI ≥ 25 kg/m^2^), while 66.9% (n = 1584) had a normal weight (BMI < 25 kg/m^2^). The highest prevalence of overnutrition was observed in 2014 (35.7%), followed by 2009 (33.2%), with the lowest in 2019 (30.9%). Further details by survey year are available in Appendix A Table A1.

In the pooled dataset, marital status was significantly associated with body weight (*p* = 0.006), with the majority of female participants being single (64.5%, n = 1521). This trend persisted in 2019, with 63.2% (n = 599, *p* = 0.024), and in 2014, when 63.9% (n = 480, *p* = 0.030) were single. Education also emerged as a key factor associated with BMI (*p* = 0.015). Overnutrition was most prevalent among women with only primary education (19.28%, n = 456), compared to those with secondary (9.4%, n = 223) or tertiary (4.4%, n = 103) education. Income level showed a significant relationship with body weight in the pooled analysis (*p* = 0.041). Women in the lowest income quintile (1st) had the highest rate of overnutrition (8.6%, n = 203), with prevalence decreasing in higher income groups, reaching the lowest in the 5th quintile (4.0%, n = 94). A similar pattern was observed in 2014, where 17.7% (n = 133) of women in the lowest income group were overnourished, compared to only 1.3% (n = 10) in the highest group (*p* = 0.037). Financial status and area of residence were not significantly associated with BMI across different survey years. When examining lifestyle factors, smoking status was significantly linked to overnutrition (*p* < 0.001), with a much higher prevalence among smokers (28.1%, n = 656) compared to non-smokers (4.8%, n = 112). This strong association persisted throughout all years. In 2019, 24.6% of smokers (n = 235) were overnourished (*p* < 0.001). Similarly, in 2014, the overnutrition rate among smokers was 32.2% (n = 242, *p* = 0.047), and in 2009, it was 28.4% (n = 179, *p* = 0.004). Overall, 57.6% (n = 1357) of participants were categorized as alcohol consumers. The highest proportion of drinkers was in 2009 (66.8%, n = 432), followed by 2014 (56.4%, n = 423), and the lowest in 2019 (52.5%, n = 502). Despite these variations across survey years, alcohol consumption was not associated with BMI. Analyzing health-related conditions showed a consistent and significant relationship between diabetes and body weight status across all survey years and the pooled dataset (*p* < 0.001). Women without diabetes had a higher prevalence of overnutrition (29.5%, n = 697) compared to those with diabetes (3.6%, n = 85). Arthrosis was less frequent among women with overnutrition. In 2009, only 13.9% reported having it (n = 92, *p* = 0.003), followed by 15.5% in 2014 (n = 116, *p* = 0.007), and 11.6% in 2019 (n = 106, *p* = 0.004). Overall, 47.3% of participants reported having arthrosis (n = 1117, *p* < 0.001). However, among women with a BMI of 25 kg/m^2^ or higher, the prevalence was lower at 13.3% (n = 314). A significant association was found between hypercholesterolemia and body weight status within the pooled sample (*p* < 0.001), with 45.6% of participants diagnosed with the condition. Among these, 7.0% (n = 163) of female participants were classified as overnourished, compared to 26.3% (n = 617) of those without the condition. Similar significant results appeared in 2009 (*p* = 0.016; 25.9%, n = 170) and in 2019 (*p* = 0.003; 27.4%, n = 258). Although women with cardiovascular disease had a slightly lower rate of overnutrition (15.2%, n = 100) than those without the condition (18.1%, n = 119), the difference was not statistically significant (*p* = 0.376). Similarly, a higher body mass index was less common among women with peptic ulcers (5.2%, n = 34) compared to those without (28.1%, n = 185). Mental illness, reported by 7.9% of participants, was significantly associated with BMI status in the pooled dataset (*p* = 0.035). Self-perceived health, which was rated as average by 50% of participants, showed no statistically significant association.

### 3.2. Factors Associated with Overnutrition

The logistic regression analysis, as shown in Table 2, identified several key predictors of overnutrition (BMI ≥ 25 kg/m^2^) among the elderly female population across individual survey years and in the pooled sample. Relationship status was a significant predictor, with married or partnered individuals exhibiting lower odds of overnutrition compared to single participants (OR = 0.77, 95% CI = 0.63–0.94, *p* = 0.011). A similar association was observed in 2019 (0.69, [0.50–0.97], *p* = 0.032). Educational level was a consistent predictor of overnutrition. Women with secondary education had higher odds than those with primary education (1.34, [1.07–1.68], *p* = 0.011), and the odds were even higher for tertiary education (1.62, [1.18–2.22], *p* = 0.003). Similar effects appeared in year-specific analyses, in 2009 (2.50, [1.19–5.26], *p* = 0.015), and in 2019 (1.52, [1.06–2.21], *p* = 0.024). Higher income quintiles seemed protective against overnutrition; women in the second (0.67, [0.51–0.88], *p* = 0.004) and third quintiles (0.76, [0.57–0.99], *p* = 0.045) had lower odds than those in the lowest quintile. In 2014, this trend was strongest, with the highest quintile associated with a 75% reduction in risk (0.25, [0.11–0.58], *p* = 0.001). Interestingly, that year, women reporting poor financial status also had a significantly lower risk of overnutrition than those with average financial status (0.58, [0.36–0.91], *p* = 0.019). Urban residence appeared protective in 2009, with women living in urban areas having 37% lower odds of overnutrition (0.63, [0.42–0.94], *p* = 0.026), although this association was not observed in later survey years. Smokers were about half as likely to be overweight compared to non-smokers (0.51, [0.38–0.68], *p* < 0.001). This effect was also evident in 2009, when smokers had a 62% lower likelihood of excess weight (0.38, [0.20–0.72], *p* = 0.003), and in 2019 (0.46, [0.31–0.69], *p* < 0.001). Several chronic conditions were associated with lower odds of overnutrition, consistent across years. Women with diabetes were more than 50% less likely to be overweight (0.46, [0.35–0.60], *p* < 0.001), and arthrosis decreased the likelihood by nearly 40% (0.62, [0.51–0.76], *p* < 0.001). Hypercholesterolemia also lowered the odds of excess weight (0.71, [0.57–0.89], *p* = 0.003). These associations were evident in individual survey years, with diabetes and arthrosis consistently protective in 2009, 2014, and 2019, while hypercholesterolemia was significant only in 2009 (0.56, [0.36–0.88], *p* = 0.012). In contrast to the year-specific findings, having a mental condition was a significant risk factor for overnutrition (1.46, [1.05–2.05], *p* = 0.026). Women who rated their health as poor had a 45% higher risk of overnutrition compared to those reporting average health (1.45, [1.15–1.82], *p* = 0.002). In 2019, this association was strong, with poor health ratings linked to more than twice the risk of overweight (2.12, [1.42–3.17], *p* < 0.001).

## 4. Discussion

This study examined the associations between socio-demographic characteristics, lifestyle behaviors, and health-related conditions and overnutrition among Hungarian women aged 65 years and older. Significant relationships were observed with marital status, educational attainment, income level, and smoking, as well as with chronic conditions such as diabetes, arthrosis, hypercholesterolemia, and mental illness. After adjustment in multivariate logistic regression models, these associations were further clarified, and self-perceived health and financial status also emerged as important determinants.

Marital status showed a significant relationship with body weight, as older women living without a partner were more likely to have a higher BMI. This finding contrasts with the common observation that being in a relationship often leads to weight gain, likely due to shared routines, dietary habits, and a sense of security that can reduce energy expenditure and physical activity [33,34,35]. However, interpretation in this study is complicated by the classification of all unmarried women, including widowed and divorced participants, into a single group. Moreover, the “crisis mode” hypothesis suggests that changes in marital status (such as divorce or losing a partner) may trigger short-term stress and weight loss [36], although such effects often diminish over time, as body weight tends to return to baseline [37]. Furthermore, age is likely to influence these relationships, with younger and older women potentially reacting differently to marital transitions. Evidence from postmenopausal women supports this idea, showing that decreases in BMI after separation or widowhood do not significantly impact long-term weight in this age group [19]. European studies further show that older women living alone are more likely to simplify their meals and follow less nutritious diets, while those living with a companion in later life often develop healthier routines and feel a stronger sense of responsibility for the household [38,39]. Previous research that combined people who were never married, divorced, or widowed found mixed results, with some showing a higher BMI in these groups [19,40]. Therefore, conducting more detailed subgroup analyses may provide further insights, as the effects of different marital histories could differ considerably.

Education level emerged as another important factor, as higher educational attainment was linked to an increased risk of overweight in older women. This contrasts with findings from many developed countries, where women with lower education levels are more likely to be obese [17,41]. Although seemingly contradictory, the pattern is more consistent with evidence from developing or less urbanized settings, as reported in the World Health Organization World Health Survey [42]. However, when examining gender and age, the South Korean study by Chung and Kim (2020) found that, while higher education was associated with lower BMI in women younger than 66, this relationship reversed in older age, with higher body weight among those with greater educational attainment [43]. Research from high-income countries further suggests that this shift reflects life-course dynamics, where highly educated women may place greater emphasis on thinness earlier in life for social and professional reasons, but decreasing social pressure in later life reduces the motivation to maintain a lower weight [44].

Nevertheless, education should be interpreted cautiously, as it often serves only as a proxy for socioeconomic status and does not fully capture differences in material resources, health literacy, or social capital that also influence body weight [45]. By contrast, income provides a more direct measure of access to resources. In this study, higher income groups were associated with a lower risk of overnutrition compared with those in the lowest income group, indicating a protective effect. These findings align with evidence from the EPIC-Norfolk cohort, where women in lower-income categories showed a higher risk of obesity after age 50 [46]. Individuals with limited income and education are more likely to face health disparities, elevated psychological stress, and increased consumption of energy-dense, nutrient-poor foods [47,48]. Still, income is not a universally reliable predictor, as associations vary across age groups, countries, and socioeconomic contexts [49]. Data from 2019 indicated that women who rated their financial situation as low were less likely to have a high BMI than those who described their situation as average. This counterintuitive finding may reflect the limitations of self-reported measures, which are influenced by subjective perceptions and may not accurately represent the actual financial resources available to older adults, who often rely on savings to cover expenses [38]. Moreover, in older age, diet largely reflects lifelong habits, remaining stable after 50, though individual differences persist beyond socioeconomic status [38]. The place of residence also influences the likelihood of being overweight. According to the 2019 survey, women residing in urban areas had a lower risk of being overweight, consistent with findings from 20 European countries, including Hungary, where overweight and obesity were more prevalent in rural regions [50]. These disparities may reflect barriers faced by older adults in rural settings, such as limited transportation options and reduced access to grocery stores and food outlets [18]. In addition to socioeconomic and residential factors, smoking status was also significantly associated with body mass index. Current smokers were less likely to be overweight compared to former or never smokers, consistent with previous research [51]. Nicotine may act as an appetite suppressant and increase energy expenditure, which could explain why some individuals, especially women, report using smoking as a strategy to control weight or reduce food cravings [52]. Given the older age group and the high prevalence of chronic illnesses, the strong protective effect of smoking observed can also be explained by reverse causality, as illness-related weight loss is common among long-term smokers in later life [53].

While alcohol consumption is culturally embedded in Hungarian dietary traditions, it did not show a significant association with overnutrition in any survey year. This pattern aligns with findings in adults 50 years and older, where alcohol intake generally decreases due to health conditions, medication use, and changes in social routines [54]. When examining both drinking patterns and the types of beverages typically consumed, studies suggest that moderate alcohol intake has no significant impact on body weight in older adults, since they tend to drink lower-calorie beverages in smaller amounts [54,55].

Several chronic conditions, including diabetes, hypercholesterolemia, and arthrosis, have been identified as protective factors associated with a lower likelihood of overnutrition. Although such findings might suggest a beneficial role, previous research indicates that this pattern is more likely to be explained by reverse causality and the so-called “obesity paradox,” in which disease-related weight loss and survival bias contribute to apparently favorable outcomes among overweight or obese older adults [56]. In cross-sectional analyses, causal inferences cannot be drawn, and weight reduction following diagnosis and treatment is common. For example, Donnelly et al. reported that older adults, particularly women, were more likely to lose weight after a diabetes diagnosis, suggesting that lower BMI reflects treatment effects or disease-related decline rather than lifelong leanness [57]. In the case of arthrosis, patients who cannot undergo total knee arthroplasty are often referred to structured programs that emphasize exercise and weight loss to reduce pain [58,59], which may account for the lower prevalence of overnutrition observed in this group. Supporting this, Yeh et al. found that fewer than 60% of older adults with knee osteoarthritis engaged in active weight control, and only one-quarter achieved a normal BMI [60]. Similarly, lifestyle interventions for hypercholesterolemia have produced concurrent improvements in LDL cholesterol and body weight, supporting the idea that diagnosis leads to lifestyle changes and subsequent BMI reduction in later life [61]. Among the chronic conditions, being diagnosed with a peptic ulcer did not show any significant association with excess weight. This is understandable given the variability in symptom presentation [40]. Some individuals eat less and may even lose weight due to pain, early fullness, or digestive discomfort that limits food intake, while others maintain a regular diet when symptoms are well controlled [40,62]. Additionally, managing ulcer symptoms primarily requires avoiding irritant foods rather than reducing total energy intake, making significant weight changes unlikely [40,62].

Similarly, no significant association was found between cardiovascular disease and excess BMI, which aligns with research indicating that the connection between body weight and CVD becomes less straightforward in older adults [63]. Many individuals with CVD experience unintentional weight loss due to disease progression, decreased appetite, medically advised dietary restrictions, and treatment side effects, all of which can obscure the expected positive relationship between higher BMI and CVD risk [53,64]. The obesity paradox described by Oreopoulos et al. provides further explanation that among older adults already diagnosed with cardiovascular disease, higher BMI is not consistently linked to worse outcomes and may, in some cases, even be associated with better survival [65].

Poor self-perceived health has also been identified as a risk factor for overnutrition. This may explain reverse causality, as individuals with obesity often rate their health more negatively, even when certain chronic conditions appear protective. Previous studies support the interpretation, showing that obese older adults consistently report poor self-rated health due to the combined burden of comorbidities and functional limitations [66]. Significantly, self-perceived health extends beyond physical functioning, as psychological and social dimensions also play a role, which may explain its strong association with body weight [67]. Consistent with this, the presence of a mental illness has been linked to a higher risk of overnutrition, as shown in several studies, including Valencio et al., who reported that women with common mental disorders were more likely to be overweight or obese, with maladaptive eating behaviors such as emotional eating and irregular dietary patterns mediating this association [29]. Moreover, this association appears to be bidirectional; mental problems can promote weight gain through overeating, physical inactivity, and side effects of psychotropic medication, while excess body weight may worsen psychological well-being by increasing stigma, social isolation, and functional decline [68].

This study has certain limitations, including its cross-sectional design and reliance on self-reported questionnaires, which may affect the accuracy of reported lifestyle behaviors and health conditions due to recall and social desirability bias. Surveying adults aged 65 and older may lead to response quality issues, as age-related memory changes or different interpretations of questions can influence how responses are provided. Since BMI was calculated from height and weight, and no objectively measured anthropometrics were available, some systematic error cannot be ruled out. These factors, along with the cross-sectional design, limit the ability to establish causality; therefore, they should be interpreted as correlations rather than causal effects, and reverse causality in chronic diseases cannot be ruled out. However, the study also has significant strengths, including the use of multivariable logistic regression and a Eurostat-validated questionnaire, which support the reliability and representativeness of the findings. An additional advantage is its focus on individuals aged 65 and older, a group often overlooked in international nutrition research despite their higher risk of obesity and chronic disease.

## 5. Conclusions

The study found that overnutrition is highly common among Hungarian women aged 65 and older, influenced by a combination of sociodemographic, lifestyle, and health-related factors. Marital status, education, income level, smoking, chronic health conditions, self-perceived health status, and mental health issues have been identified as important factors, reflecting the complex nature of body weight regulation in later life. The results highlight that social and health status contribute to obesity risk among elderly women, while conditions such as diabetes or hypercholesterolemia may seem protective due to reverse causality and changes in lifestyle after diagnosis.

Given Hungary’s high obesity rates and aging population, these findings underscore the need for targeted interventions that address socioeconomic disparities, promote healthy eating habits, encourage active lifestyles, and include mental healthcare in obesity prevention efforts. However, the cross-sectional design limits causal inference; the use of nationally representative survey data provides strong evidence on this underrepresented group in obesity research. Future longitudinal and intervention studies are needed to understand causal pathways better and develop policies to reduce obesity among older women.

## Figures and Tables

**Table 1:** Descriptive characteristics of Hungarian women aged ≥65 years according to BMI category.

Variable	Category	Body Weight		Total n (%)	*p* Value
		Normal BMI < 25	Overnutrition BMI ≥ 25		
Marital status	Single/ divorced/widowed	988 (41.9%)	533 (22.6%)	1521 (64.5%)	**0.006**
	Married/partnered	590 (25.0%)	246 (10.4%)	836 (35.5%)	
Education	Primary	1018 (43.0%)	456 (19.3%)	1474 (62.3%)	**0.015**
	Secondary	398 (16.8%)	223 (9.4%)	621 (26.2%)	
	Tertiary	167 (7.1%)	103 (4.4%)	270 (11.4%)	
Household income quintile	1st	353 (14.9%)	203 (8.6%)	556 (23.5%)	**0.041**
	2nd	387 (16.4%)	152 (6.4%)	539 (22.8%)	
	3rd	348 (14.7%)	164 (6.9%)	512 (21.6%)	
	4th	323 (13.7%)	170 (7.2%)	493 (20.9%)	
	5th	173 (7.3%)	94 (4.0%)	267 (11.3%)	
Financial status	Average	1014 (43.1%)	513 (21.8%)	1527 (64.9%)	0.599
	Higher than average	238 (10.1%)	120 (5.1%)	358 (15.2%)	
	Lower than average	321 (13.6%)	145 (6.2%)	466 (19.8%)	
Area of residence	Rural	463 (19.6%)	217 (9.2%)	680 (28.8%)	0.443
	Urban	1121 (47.4%)	566 (23.9%)	1687 (71.2%)	
Smoking status	Non-smoker	118 (5.1%)	112 (4.8%)	230 (9.9%)	**<0.001**
	Smoker	1452 (62.1%)	656 (28.1%)	2108 (90.1%)	
Alcohol use	Non-drinker	666 (28.3%)	331 (14.1%)	997 (42.4%)	0.777
	Drinker	914 (38.8%)	443 (18.8%)	1357 (57.6%)	
Diabetes	No	1227 (52.0%)	697 (29.5%)	1924 (81.5%)	**<0.001**
	Yes	353 (14.9%)	85 (3.6%)	438 (18.5%)	
Arthrosis	No	777 (33.0%)	464 (19.7%)	1241 (52.7%)	**<0.001**
	Yes	803 (34.1%)	314 (13.3%)	1117 (47.3%)	
Peptic ulcer	No	1465 (62.1%)	709 (30.1%)	2174 (92.2%)	0.110
	Yes	114 (4.8%)	71 (3.0%)	185 (7.8%)	
Hypercholesterolemia	No	1114 (47.5%)	617 (26.3%)	1731 (73.8%)	**<0.001**
	Yes	452 (19.3%)	163 (7.0%)	615 (26.2%)	
CVD	No	951 (40.2%)	472 (19.9%)	1423 (60.1%)	0.910
	Yes	633 (26.7%)	311 (13.1%)	944 (39.9%)	
Self-perceived health status	Average	811 (34.3%)	371 (15.7%)	1182 (50.0%)	0.195
	Higher than average	301 (12.7%)	166 (7.0%)	467 (19.7%)	
	Lower than average	470 (19.9%)	245 (10.4%)	715 (30.3%)	
Mental illness	No	1464 (62.1%)	706 (29.9%)	2170 (92.0%)	**0.035**
	Yes	112 (4.8%)	75 (3.2%)	187 (8.0%)	

Bold values indicate statistical significance (*p* < 0.05) based on the chi-square test.

**Table 2:** Logistic regression results for predictors of overnutrition among elderly Hungarian women by survey year and pooled sample.

Variable	Category/Level	Pooled Sample OR (95% CI)	*p*-Value	2009 OR (95% CI)	*p*-Value	2014 OR (95% CI)	*p*- Value	2019 OR (95% CI)	*p*-Value
Marital status	Single/divorced/widowed (Reference)								
	Married/partnered	0.77 (0.63–0.94)	**0.011**	0.96 (0.65–1.43)	0.855	0.83 (0.56–1.22)	0.343	0.69 (0.50–0.97)	**0.032**
Education	Primary (Reference)								
	Secondary	1.34 (1.07–1.68)	**0.011**	1.35 (0.82–2.23)	0.233	1.15 (0.77–1.71)	0.507	1.53 (1.06–2.21)	**0.024**
	Tertiary	1.62 (1.18–2.22)	**0.003**	2.50 (1.19–5.26)	**0.015**	1.49 (0.86–2.60)	0.158	1.38 (0.82–2.33)	0.224
Household income quintile	1st (Reference)								
	2nd	0.67 (0.51–0.88)	**0.004**	0.75 (0.32–1.74)	0.503	0.64 (0.41–1.01)	0.056	0.82 (0.52–1.30)	0.402
	3rd	0.76 (0.57–0.99)	**0.045**	0.97 (0.45–2.08)	0.929	0.65 (0.39–1.09)	0.104	0.92 (0.57–1.48)	0.732
	4th	0.82 (0.62–1.08)	0.150	1.00 (0.48–2.08)	0.999	0.87 (0.47–1.60)	0.654	1.01 (0.59–1.72)	0.968
	5th	0.82 (0.58–1.17)	0.277	1.30 (0.58–2.92)	0.523	0.25 (0.11–0.58)	**0.001**	1.49 (0.69–3.23)	0.311
Financial status	Average (Reference)								
	Higher than average	1.00 (0.77–1.30)	0.994	0.95 (0.50–1.80)	0.867	1.35 (0.84–2.15)	0.215	0.93 (0.62–1.38)	0.717
	Lower than average	0.82 (0.64–1.05)	0.111	1.04 (0.68–1.58)	0.869	0.58 (0.37–0.91)	**0.019**	0.88 (0.56–1.41)	0.602
Area of residence	Rural (Reference)								
	Urban	0.96 (0.78–1.19)	0.721	0.63 (0.42–0.94)	**0.026**	1.08 (0.76–1.55)	0.662	1.09 (0.76–1.56)	0.649
Smoking status	Non-smoker (Reference)								
	Smoker	0.51 (0.38–0.68)	**<0.001**	0.38 (0.20–0.72)	**0.003**	0.62 (0.34–1.14)	0.123	0.46 (0.31–0.69)	**<0.001**
Alcohol use	Non-drinker (Reference)								
	Drinker	1.05 (0.86–1.27)	0.651	1.22 (0.82–1.81)	0.336	1.20 (0.86–1.67)	0.287	0.91 (0.66–1.25)	0.568
Diabetes	No (Reference)								
	Yes	0.46 (0.35–0.60)	**<0.001**	0.46 (0.27–0.77)	**0.003**	0.44 (0.28–0.71)	**0.001**	0.47 (0.30–0.73)	**0.001**
Arthrosis	No (Reference)								
	Yes	0.62 (0.51–0.76)	**<0.001**	0.61 (0.42–0.90)	**0.011**	0.58 (0.41–0.82)	**0.002**	0.59 (0.43–0.82)	**0.002**
Peptic ulcer	No (Reference)								
	Yes	1.37 (0.98–1.92)	0.063	1.48 (0.88–2.51)	0.141	1.07 (0.58–1.98)	0.829	1.39 (0.70–2.74)	0.347
Hypercholesterolemia	No (Reference)								
	Yes	0.71 (0.57–0.89)	**0.003**	0.56 (0.36–0.88)	**0.012**	0.81 (0.55–1.19)	0.282	0.76 (0.53–1.09)	0.136
CVD	No (Reference)								
	Yes	1.12 (0.91–1.37)	0.291	1.19 (0.81–1.76)	0.383	1.27 (0.89–1.80)	0.192	0.95 (0.67–1.34)	0.761
Self-perceived health	Average (Reference)								
	Higher than average	1.01 (0.79–1.30)	0.923	0.90 (0.52–1.56)	0.719	0.93 (0.59–1.45)	0.738	1.17 (0.80–1.71)	0.429
	Lower than average	1.45 (1.15–1.82)	**0.002**	1.04 (0.68–1.60)	0.844	1.30 (0.87–1.93)	0.194	2.12 (1.42–3.17)	**<0.001**
Mental illness	No (Reference)								
	Yes	1.46 (1.05–2.05)	**0.026**	1.52 (0.64–3.58)	0.342	1.72 (0.88–3.36)	0.111	1.48 (0.93–2.34)	0.095

Bold values indicate statistical significance (*p* < 0.05). Odds ratios are adjusted for variables in the model.

## Data Availability

The data analyzed in this study is subject to the following licenses/restrictions: The data presented in this study are available upon request from Hungarian Central Statistical Office who performed and supervised the data collection. Requests to access these datasets should be directed to Hungarian Central Statistical Office, https://www.ksh.hu/?lang=en, accessed on 4 December 2025.

## Appendix A

**Table A1:** Descriptive characteristics of Hungarian women aged ≥65 years by survey year (2009, 2014, and 2019) and BMI category.

Category	Variables	2009				2014				2019			
		Normal BMI < 25, n (%)	Overnutrition BMI ≥ 25, n (%)	Total, n (%)	*p*-Value	Normal BMI < 25, n (%)	Overnutrition BMI ≥ 25, n (%)	Total, n (%)	*p*-Value	Normal BMI < 25, n (%)	Overnutrition BMI ≥ 25, n (%)	Total, n (%)	*p*-Value
Gender	Female	440 (66.8%)	219 (33.2%)	659 (100.0%)	-	483 (64.3%)	268 (35.7%)	751 (100.0%)	-	661 (69.1%)	296 (30.9%)	957 (100.0%)	-
Marital status	Single/divorced/widowed	294 (44.7%)	148 (22.0%)	442 (66.7%)	0.875	295 (39.3%)	185 (24.6%)	480 (63.9%)	**0.030**	399 (42.1%)	200 (21.1%)	599 (63.2%)	**0.024**
	Married/partnered	145 (22.5%)	71 (10.8%)	216 (33.3%)		188 (25.0%)	83 (11.1%)	271 (36.1%)		257 (27.1%)	92 (9.7%)	349 (36.8%)	
Education	Primary	339 (51.6%)	148 (22.5%)	487 (74.1%)	**0.011**	318 (42.3%)	168 (22.4%)	486 (64.7%)	0.617	361 (37.7%)	140 (14.6%)	501 (52.4%)	0.102
	Secondary	75 (11.4%)	45 (6.9%)	120 (18.3%)		115 (15.3%)	67 (8.9%)	182 (24.2%)		208 (21.7%)	111 (11.6%)	319 (33.1%)	
	Tertiary	25 (3.8%)	25 (3.8%)	50 (7.6%)		50 (6.7%)	33 (4.4%)	83 (11.1%)		92 (9.6%)	45 (4.7%)	253 (26.4%)	
Household income quintile	1st	33 (5.0%)	16 (2.4%)	49 (7.4%)	0.397	197 (26.2%)	133 (17.7%)	330 (44.0%)	**0.037**	123 (12.9%)	54 (5.6%)	137 (14.3%)	0.125
	2nd	61 (9.3%)	22 (3.3%)	83 (12.6%)		110 (14.7%)	54 (7.2%)	164 (21.8%)		216 (22.6%)	76 (7.9%)	177 (18.5%)	
	3rd	102 (15.5%)	48 (7.3%)	150 (22.8%)		87 (11.6%)	43 (5.7%)	130 (17.3%)		159 (16.6%)	73 (7.6%)	292 (30.5%)	
	4th	148 (22.5%)	72 (10.9%)	220 (33.4%)		47 (6.3%)	28 (3.7%)	75 (10.0%)		128 (13.4%)	70 (7.3%)	232 (24.2%)	
	5th	96 (14.6%)	61 (9.3%)	157 (23.8%)		42 (5.6%)	10 (1.3%)	52 (6.9%)		35 (3.7%)	23 (2.4%)	198 (20.0%)	
Financial status	Average	266 (40.5%)	134 (20.4%)	400 (60.7%)	0.780	314 (42.0%)	184 (24.6%)	498 (66.6%)	0.159	434 (45.9%)	195 (20.6%)	629 (66.5%)	0.862
	Higher than average	34 (5.2%)	20 (3.0%)	54 (8.2%)		70 (9.4%)	44 (5.9%)	114 (15.2%)		134 (14.2%)	56 (5.9%)	190 (20.1%)	
	Lower than average	138 (21.0%)	65 (9.9%)	203 (30.9%)		97 (13.0%)	39 (5.2%)	136 (18.2%)		86 (9.1%)	41 (4.3%)	127 (13.4%)	
Area of residence	Rural	136 (20.6%)	71 (10.8%)	207 (31.4%)	0.694	146 (19.4%)	76 (10.1%)	222 (29.6%)	0.591	181 (18.9%)	70 (7.3%)	251 (26.2%)	0.225
	Urban	304 (46.1%)	148 (22.5%)	452 (68.6%)		337 (44.9%)	192 (25.6%)	529 (70.4%)		480 (50.2%)	226 (23.6%)	706 (73.8%)	
Smoking status	Non-smoker	24 (3.8%)	25 (4.0%)	49 (7.8%)	**0.004**	28 (3.7%)	26 (3.5%)	54 (7.2%)	**0.047**	66 (6.9%)	61 (6.4%)	127 (13.3%)	**<0.001**
	Smoker	402 (63.8%)	179 (28.4%)	581 (92.2%)		455 (60.6%)	242 (32.2%)	697 (92.8%)		595 (62.2%)	235 (24.6%)	830 (86.7%)	
Alcohol use	Non-drinker	143 (22.1%)	72 (11.1%)	215 (33.0%)	0.737	217 (28.9%)	110 (14.7%)	327 (43.6%)	0.324	306 (32.0%)	149 (15.6%)	455 (47.5%)	0.247
	Drinker	293 (45.3%)	139 (21.5%)	432 (66.8%)		266 (35.5%)	157 (20.9%)	423 (56.4%)		355 (37.1%)	147 (15.4%)	502 (52.5%)	
Diabetes	No	336 (51.0%)	193 (29.3%)	529 (80.3%)	**<0.001**	378 (50.3%)	240 (32.0%)	618 (82.3%)	**<0.001**	513 (53.9%)	264 (27.7%)	777 (81.6%)	**<0.001**
	Yes	104 (15.8%)	26 (4.0%)	130 (19.8%)		105 (14.0%)	28 (3.7%)	133 (17.7%)		144 (15.1%)	31 (3.3%)	175 (18.4%)	
Arthrosis	No	201 (30.5%)	127 (19.3%)	328 (49.8%)	**0.003**	223 (29.8%)	151 (20.2%)	374 (49.9%)	**0.007**	353 (37.2%)	186 (19.6%)	539 (56.7%)	**0.004**
	Yes	239 (36.3%)	92 (14.0%)	331 (50.2%)		259 (34.6%)	116 (15.5%)	375 (50.1%)		305 (32.1%)	106 (11.2%)	411 (43.3%)	
Peptic ulcer	No	384 (58.3%)	185 (28.1%)	569 (86.3%)	0.325	452 (60.3%)	247 (32.9%)	699 (93.2%)	0.576	629 (66.2%)	277 (29.2%)	906 (95.4%)	0.259
	Yes	56 (8.5%)	34 (5.2%)	90 (13.7%)		31 (4.1%)	20 (2.7%)	51 (6.8%)		27 (2.8%)	17 (1.8%)	44 (4.6%)	
Hypercholesterolemia	No	311 (47.4%)	175 (26.7%)	486 (73.7%)	**0.016**	352 (47.1%)	209 (27.9%)	561 (75.0%)	0.094	451 (47.9%)	233 (24.7%)	684 (72.6%)	**0.003**
	Yes	126 (19.2%)	44 (6.7%)	170 (25.8%)		130 (17.4%)	57 (7.6%)	187 (25.0%)		196 (20.8%)	62 (6.6%)	258 (27.4%)	
CVD	No	223 (33.8%)	119 (18.1%)	342 (51.9%)	0.376	297 (39.6%)	154 (20.5%)	451 (60.1%)	0.280	431 (45.0%)	199 (20.8%)	630 (65.8%)	0.541
	Yes	217 (32.9%)	100 (15.2%)	317 (48.1%)		186 (24.8%)	114 (15.2%)	300 (39.9%)		230 (24.0%)	97 (10.1%)	327 (34.2%)	
Self-perceived health status	Average	200 (30.4%)	104 (15.8%)	304 (46.1%)	0.225	247 (32.9%)	127 (16.9%)	374 (49.8%)	0.567	364 (38.2%)	140 (14.7%)	504 (52.8%)	0.077
	Higher than average	61 (9.3%)	39 (5.9%)	100 (15.2%)		97 (12.9%)	55 (7.3%)	152 (20.2%)		143 (15.0%)	72 (7.6%)	215 (22.5%)	
	Lower than average	179 (27.2%)	76 (11.5%)	255 (38.7%)		139 (18.5%)	86 (11.5%)	225 (30.0%)		152 (15.9%)	83 (8.7%)	235 (24.6%)	
Mental illness	No	421 (63.9%)	207 (31.4%)	628 (95.3%)	0.507	460 (61.3%)	247 (32.9%)	707 (94.1%)	0.086	583 (61.6%)	252 (26.6%)	835 (88.2%)	0.116
	Yes	19 (2.9%)	12 (1.8%)	31 (4.7%)		23 (3.1%)	21 (2.8%)	44 (5.9%)		70 (7.4%)	42 (4.4%)	112 (11.8%)	

Bold values indicate statistical significance (*p* < 0.05) based on the chi-square test.
